# Supplementary material for: Neuron tracing and quantitative analyses of dendritic architecture reveal symmetrical three-way-junctions and phenotypes of git-1 in C. elegans
Source: PLoS Comput Biol. 2021 Jul 19;17(7):e1009185. doi: 10.1371/journal.pcbi.1009185 (PMC8321406; doi:10.1371/journal.pcbi.1009185)
Supplement: S1 Fig — This architecture is made of an encoder subunit through which the input image shrinks in size, and a decoder subunit through which the image is upsampled back to its original size. The input layer gets 64x64 pixels patches of grayscale neuronal images and applies zero-centering normalization to them. Then, two convolution units (blue squares) are applied, with each unit consisting of a 2D 3x3 convolution layer, followed by a batch normalization layer (BN) and a rectified linear unit (ReLU) layer. This unit is followed by a 2x2 max pooling layer (green square) that reduces the size of the input by a factor of four. This sequence is repeated three times in the encoder, resulting in a 8x8 feature map with 64 features. The same sequence is repeated in the decoder, but with each max pooling layer replaced by an upsampling layer (purple square) that precedes each sequence of convolutions. Numbers below encoder layers show the output size of the pooling layer, and in the decoder they show the input to the upsampling layer. Finally, the softmax layer (yellow square) takes the output of the last ReLU layer and converts it into a probability distribution that sums up to 1. The last layer of the network is a pixel classification layer (red square). This layer computes the loss (cross-entropy) during training and performs the prediction of one of the predefined classes for new data. (PDF) [file pcbi.1009185.s001.pdf]

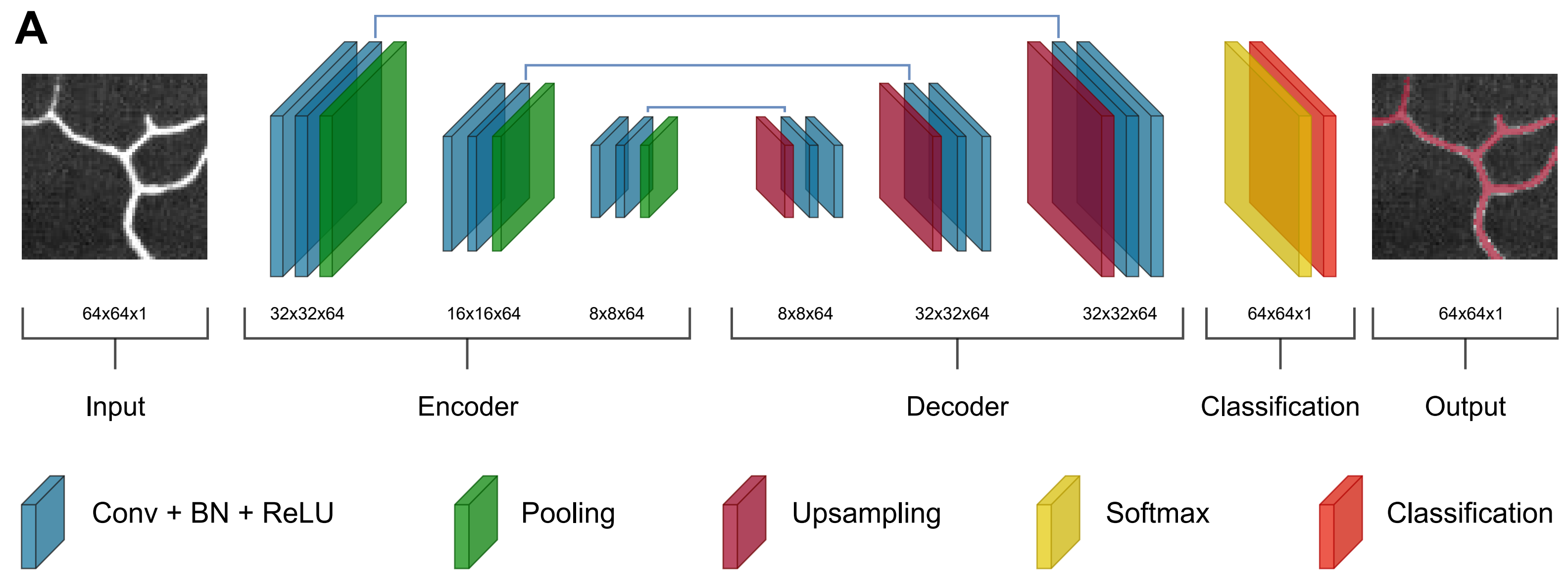
A
64x64x1
32x32x64
16x16x64
8x8x64
8x8x64
32x32x64
32x32x64
64x64x1
64x64x1
Input
Encoder
Decoder
Classification
Output
Conv + BN + ReLU
Pooling
Upsampling
Softmax
Classification
