# Supplementary figures and images for: Neuron tracing and quantitative analyses of dendritic architecture reveal symmetrical three-way-junctions and phenotypes of git-1 in C. elegans
Source: PLoS Comput Biol. 2021 Jul 19;17(7):e1009185. doi: 10.1371/journal.pcbi.1009185 (PMC8321406; doi:10.1371/journal.pcbi.1009185)

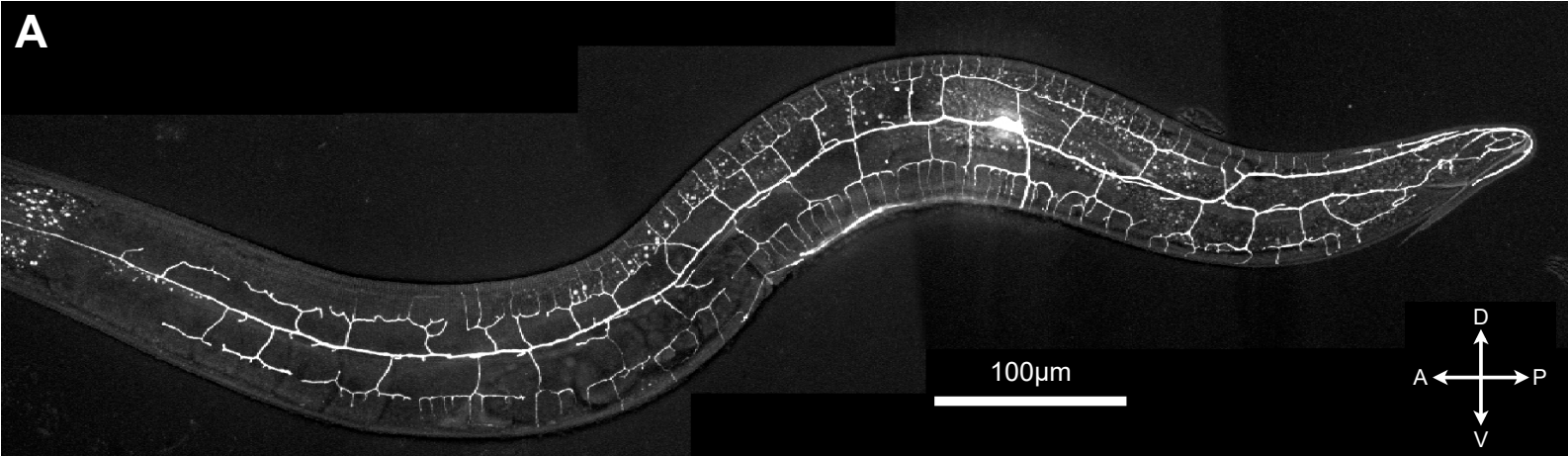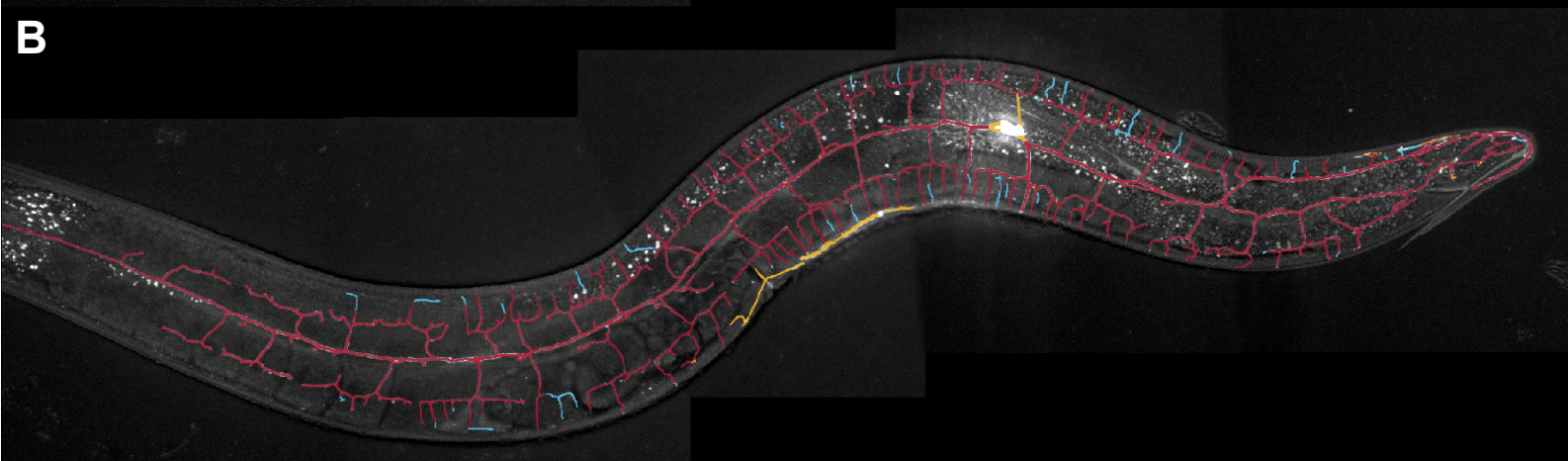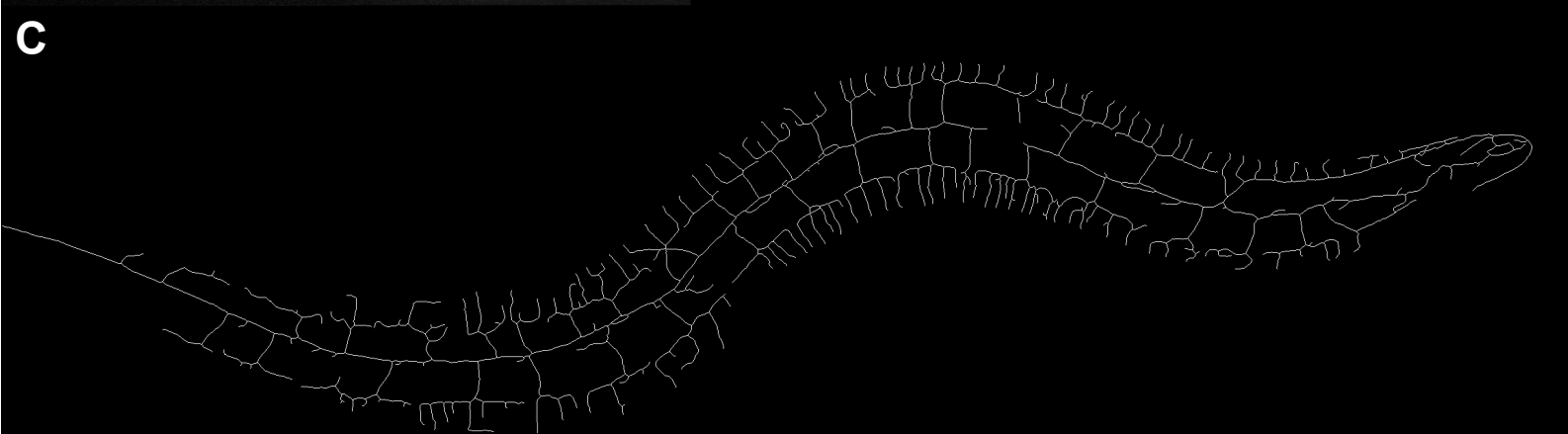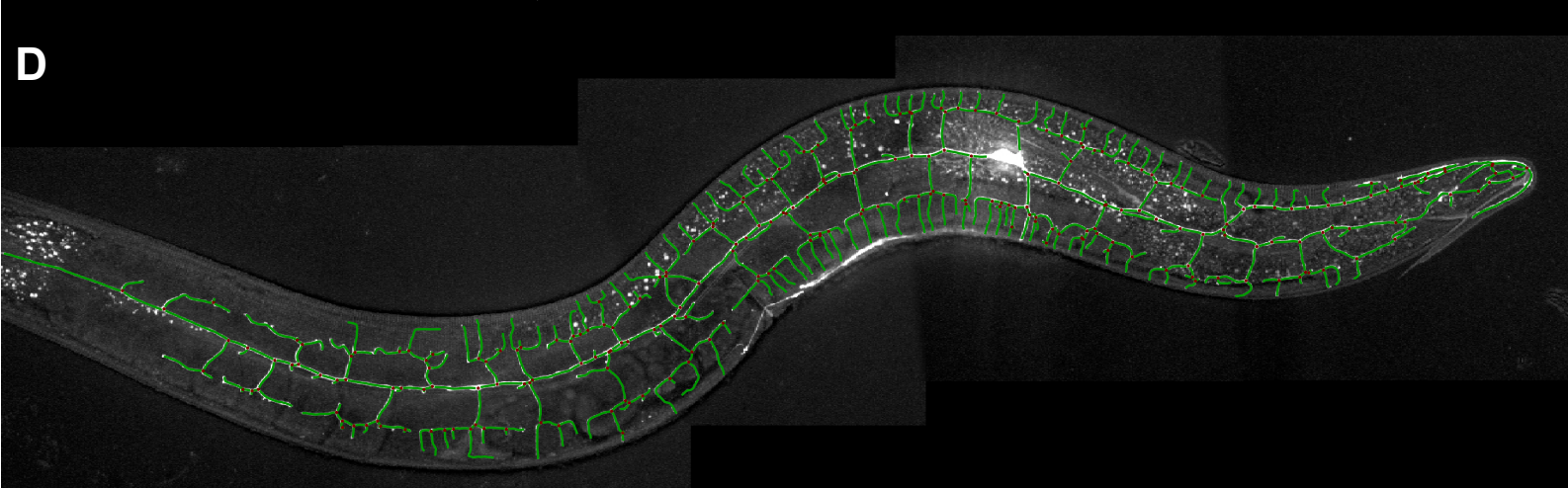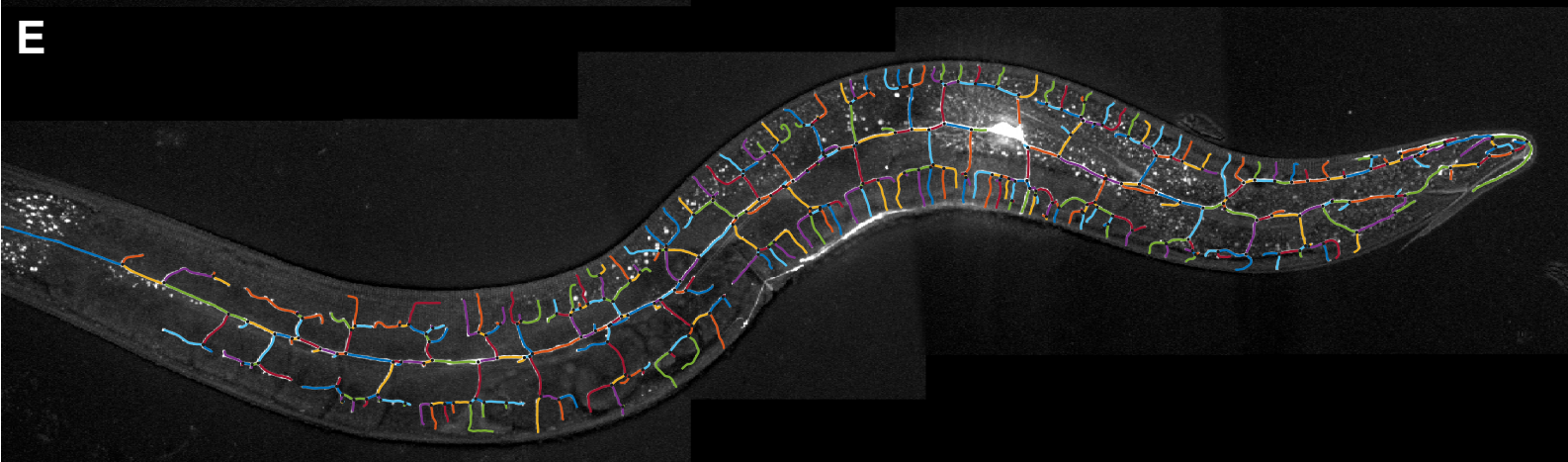

Supplement: S2 Fig — A. A maximum intensity, grayscale image of a PVD neuron. Arrows show the anterior (A), posterior (P), dorsal (D) and ventral (V) directions. B. The classification of the image into neuron and non-neuron pixels. CNN derived classification appears in red, manually added pixels in blue and manually removed pixels in yellow. C. Skeleton image derived from the binary image in B. D. The fully traced neuron. E. Segmentation of the traced neuron. Different segments appear in different colors. (PDF) [file pcbi.1009185.s002.pdf]

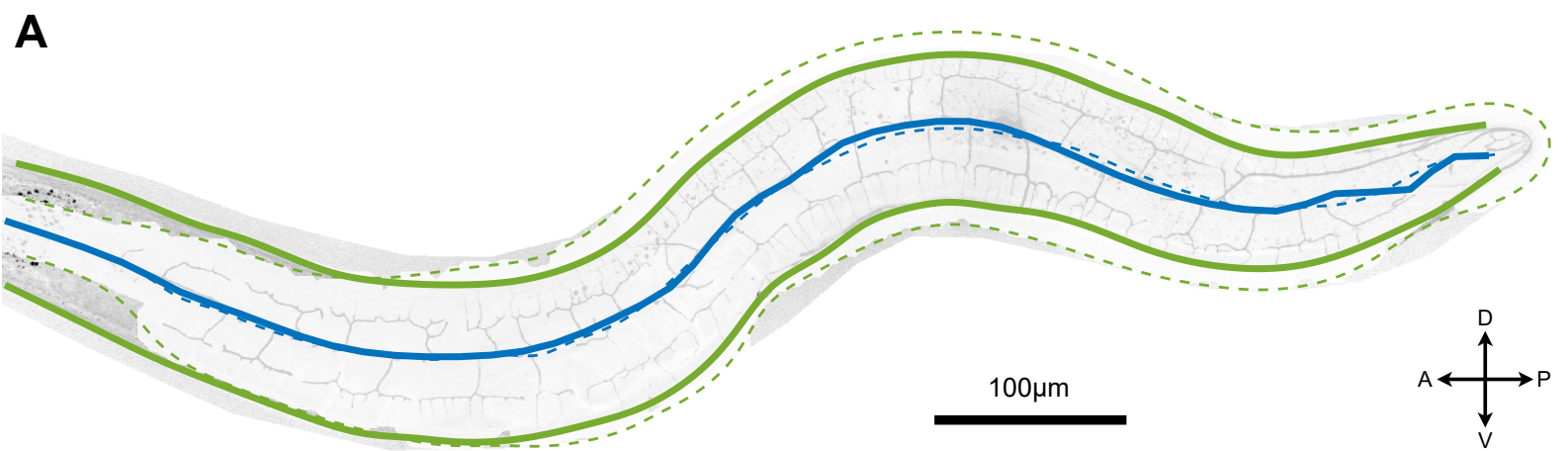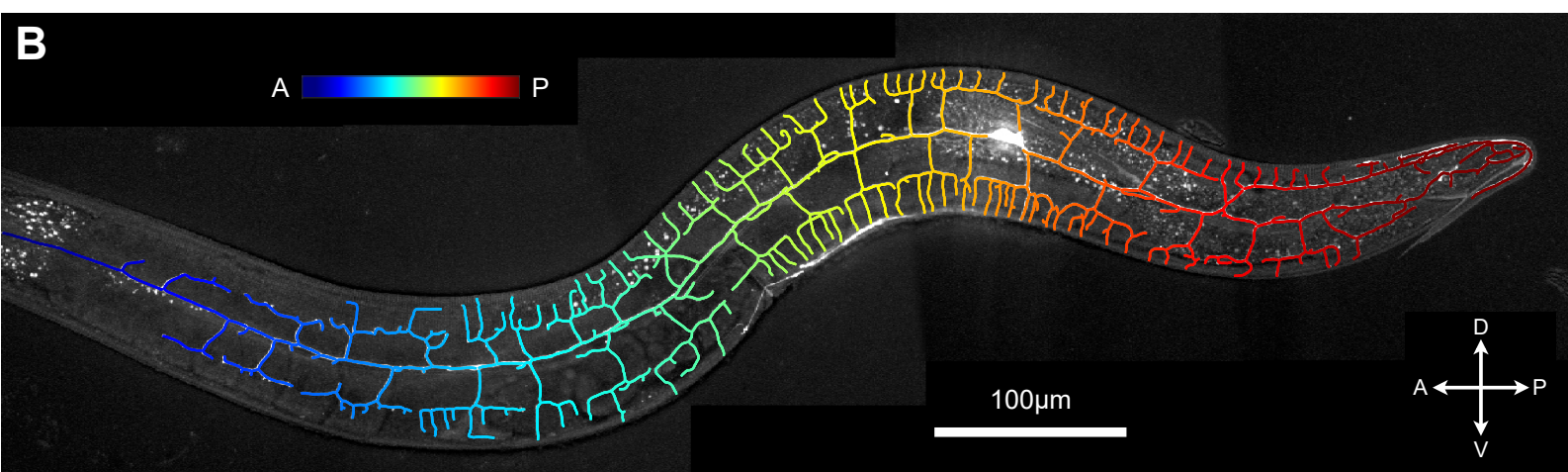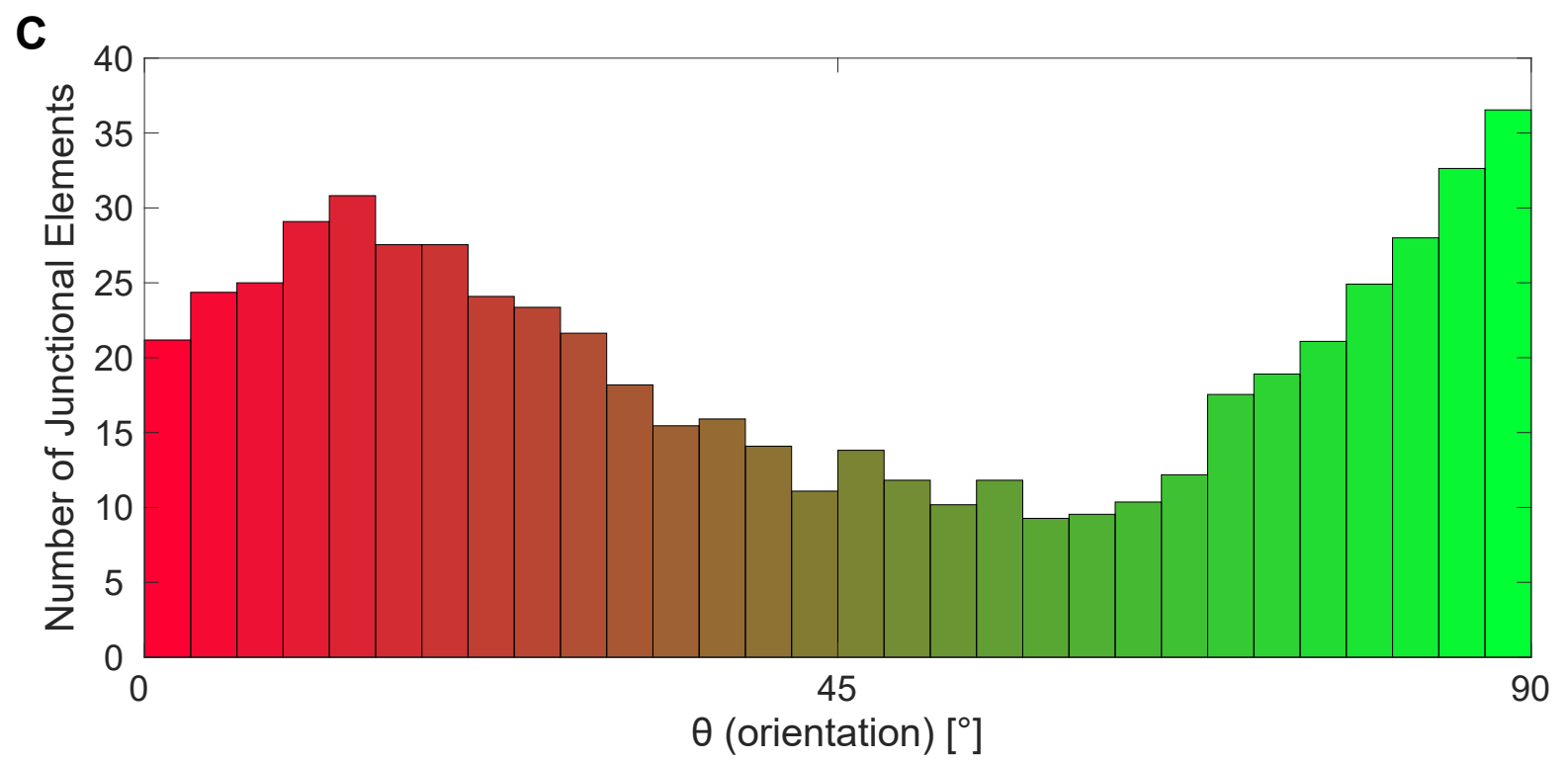

Supplement: S3 Fig — A. The neuron’s trace is used to generate a single blob image. Then, the centerline and boundary of the blob are detected (dashed blue and green lines respectively). These are then refined using a sliding window along the approximated centerline, resulting in the final midline and boundary of the neuron (solid lines). B. A PVD color-coded for midline coordinate, from anterior (A) to posterior (P). Each neuron element is associating with a midline point by shortest distance, and colored according to its corresponding midline arclength from anterior to posterior. C. The distribution of midline orientation of junction rectangles. This distribution is different from the one of all neuron elements (Fig 3H), with peaks at 13.5° and 90°. (PDF) [file pcbi.1009185.s003.pdf]

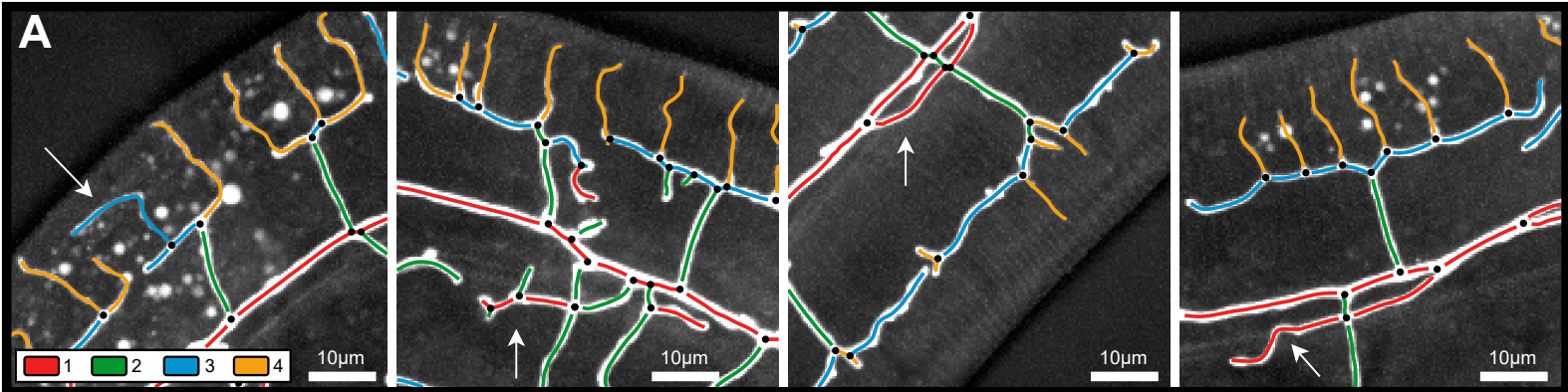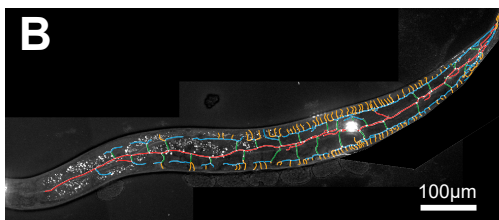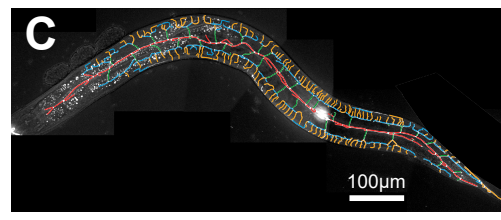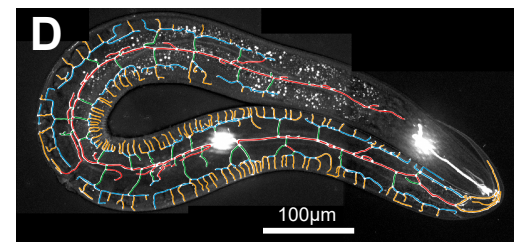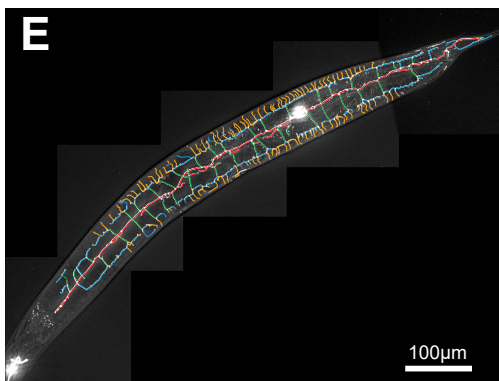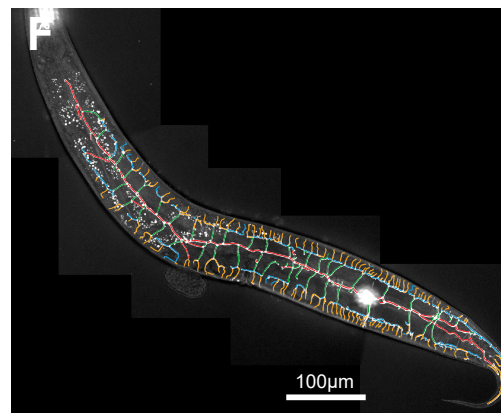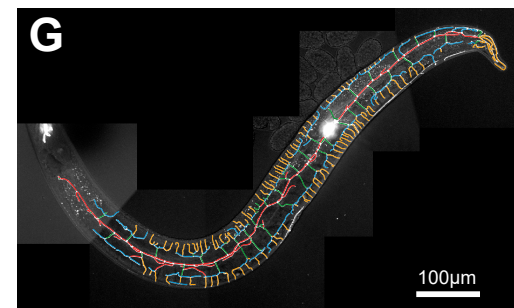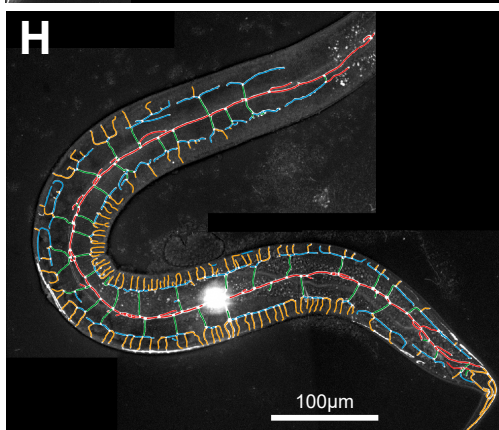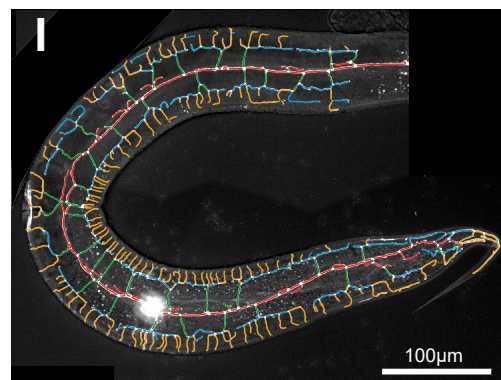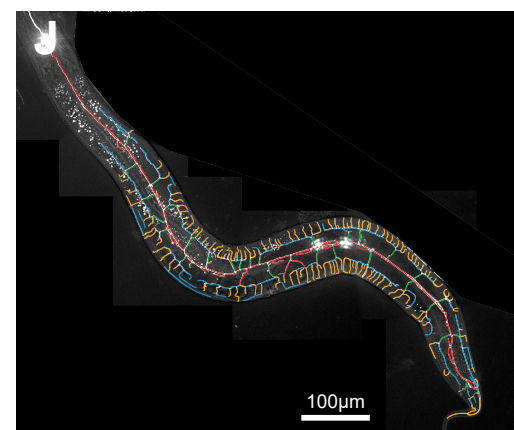

Supplement: S4 Fig — Classes color-coded as in Fig 4A–4E: Class 1 (red), class 2 (green), class 3 (blue) and class 4 (yellow). A. Magnified regions of wild-type PVDs algorithmically classified into morphological classes. Arrows show classifications that do not match the conventional manual classification into Menorah orders. B-J. Visualization of the algorithmically-derived classification in nine PVD images of wild-type C. elegans worms (in addition to the one in Fig 4C). (PDF) [file pcbi.1009185.s004.pdf]

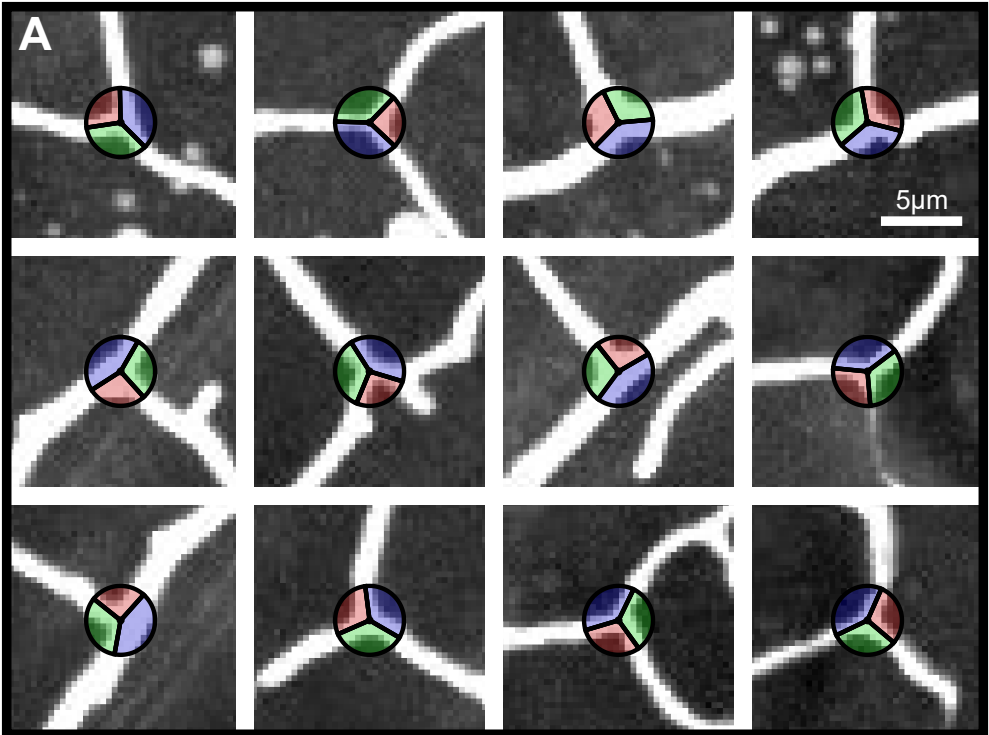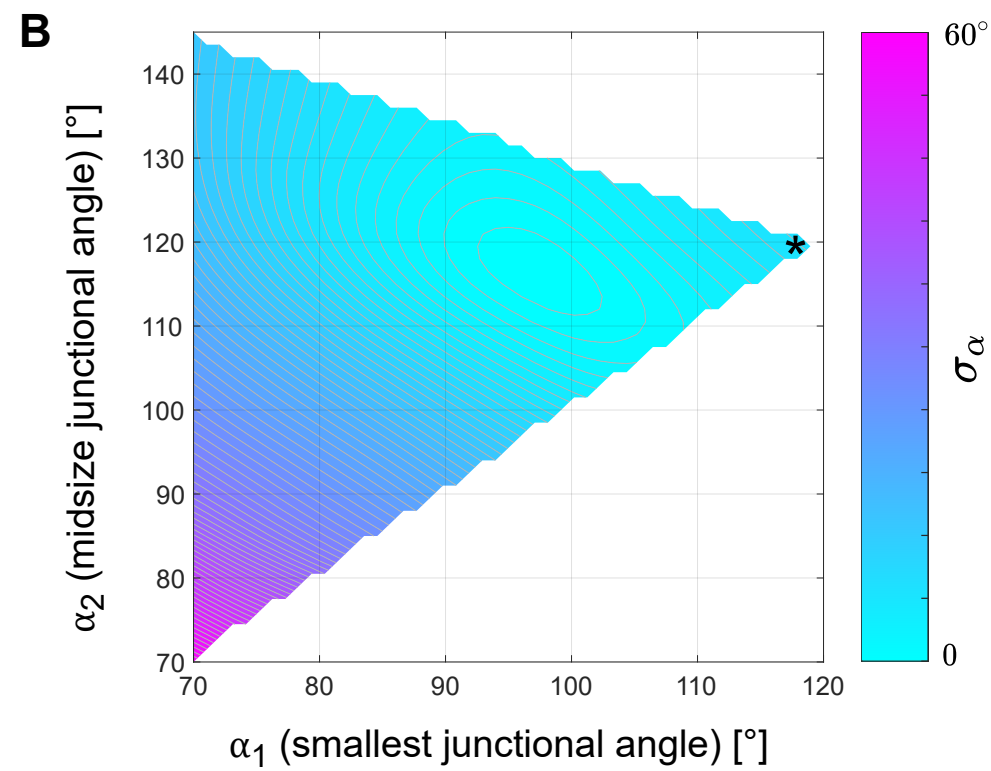

Supplement: S5 Fig — A. Examples of PVD junction morphologies, showing a two-times larger neighborhood compared with Fig 5A, to demonstrate that process orientation often does not match junctional angles. Colors correspond to relative angle size: smallest (red), mid-size (green) and largest (blue). B. The variability in junction geometries is characterized by angular noise, determined from a Monte-Carlo simulation. Fit with simulated distributions gives a junction variability of 18° around the symmetrical configuration (120°-120°-120°), as indicated by the star symbol. (PDF) [file pcbi.1009185.s005.pdf]

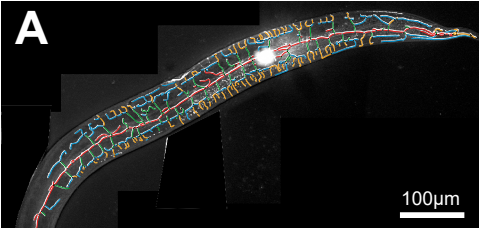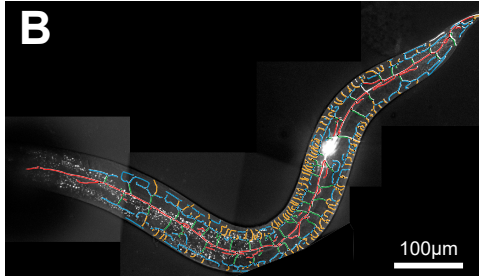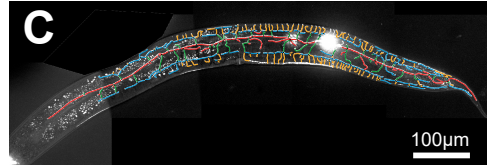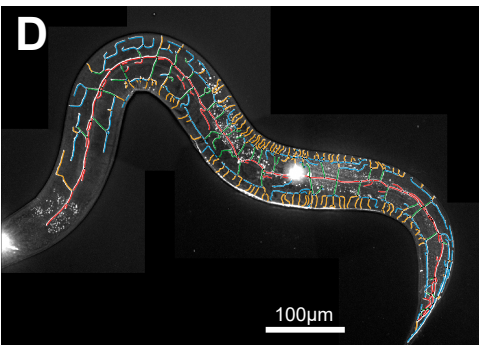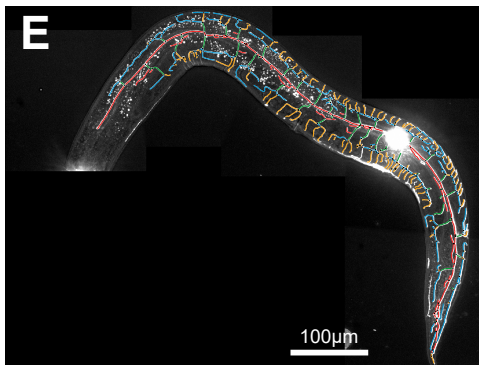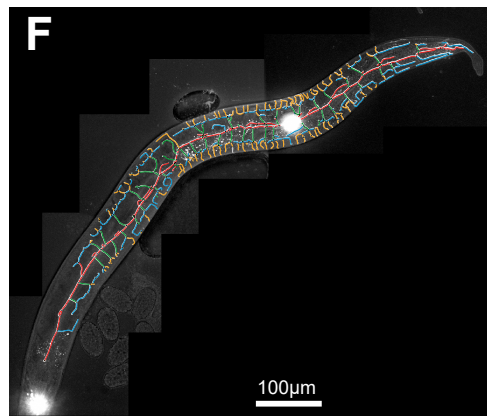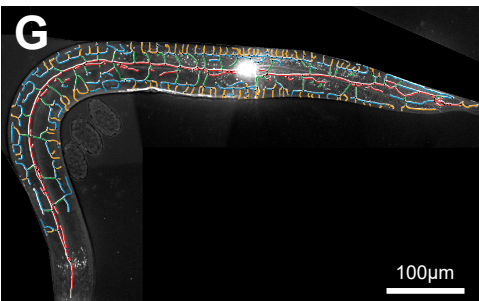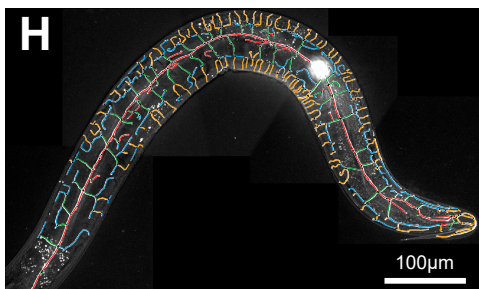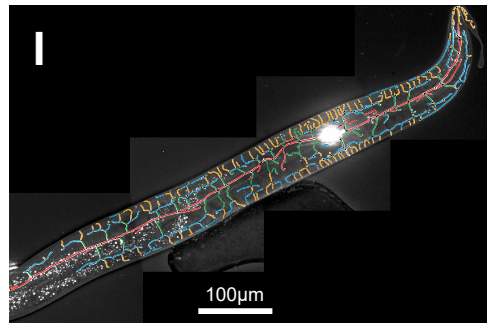

Supplement: S6 Fig — A-I. Visualization of the algorithmically-derived classification in nine PVD images of git-1(ok1848) mutants (in addition to the one in Fig 6A). (PDF) [file pcbi.1009185.s006.pdf]

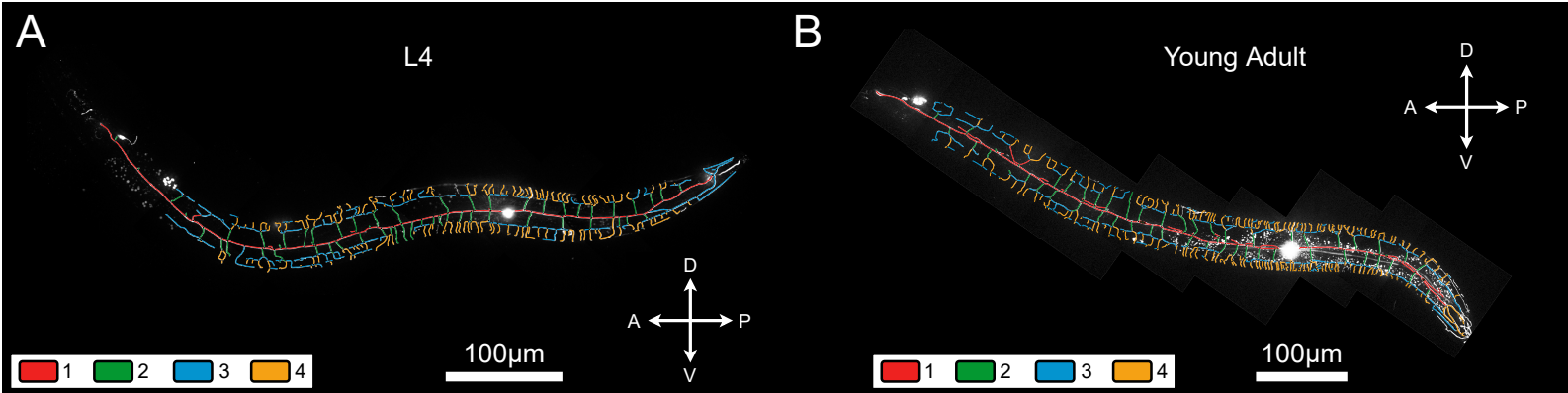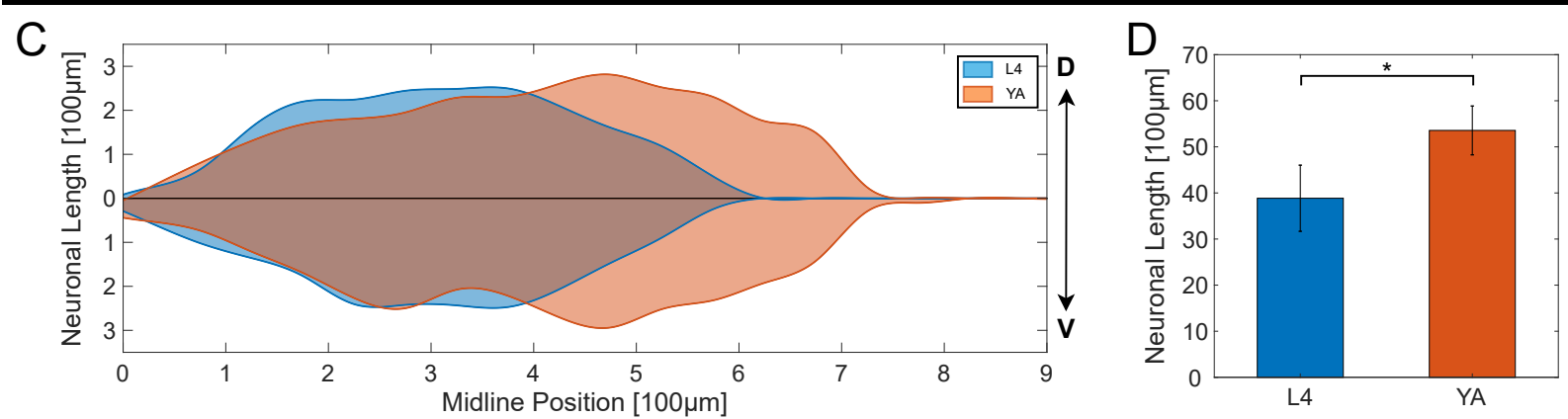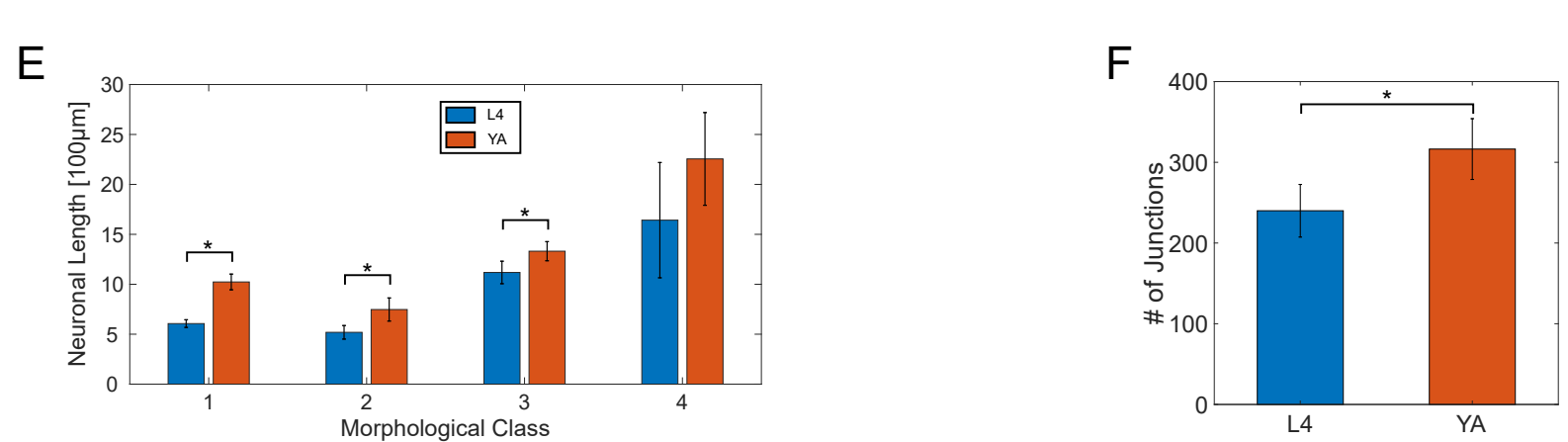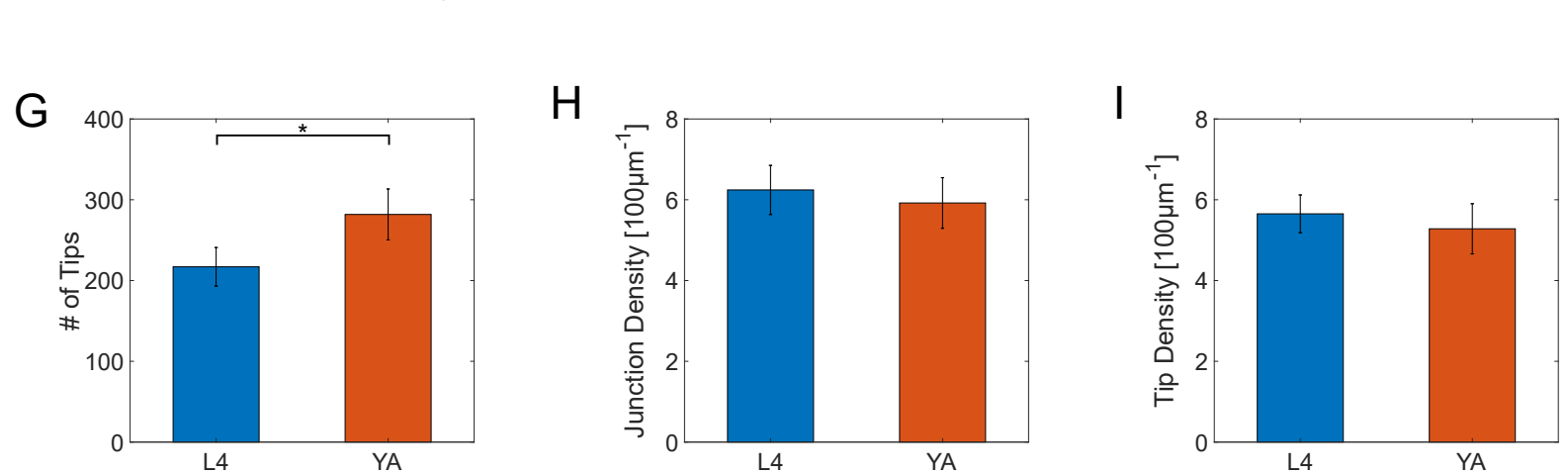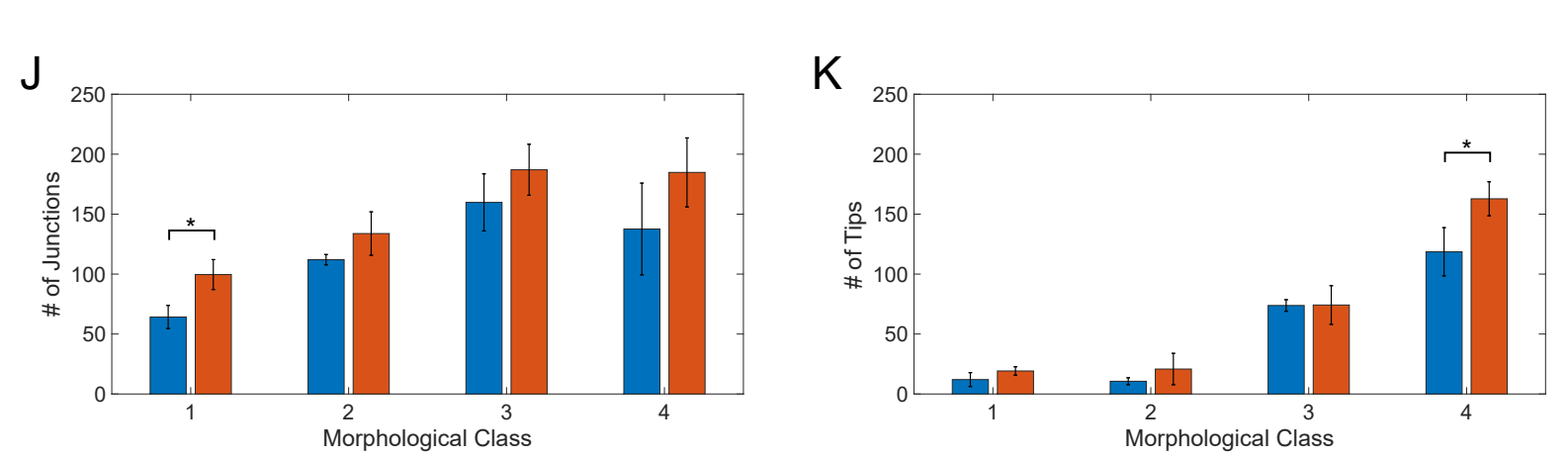

Supplement: S7 Fig — A. An image of a full PVD of an L4 worm, superimposed with color-coded morphological classes: class 1 (red), class 2 (green), class 3 (blue), class 4 (yellow). B. An image of a full PVD of a young-adult worm, superimposed with color-coded morphological classes, as in A. C. Distribution of neuronal length along the midline (head = 0), averaged across worms, for L4 (blue, n = 5) and young-adult (red, n = 5) worms. D. The total PVD length for L4 (blue) and young-adult (red) worms. E. The total PVD length for each morphological class for L4 (blue) and young-adult (red) worms. F. The total number of dendritic junctions, as described in D. G. The total number of dendritic tips, as described in D. H. The density of dendritic junctions, as described in D. I. The density of dendritic tips, as described in D. J. The total number of dendritic junctions for each morphological, as described in E. K. The total number of dendritic tips for each morphological, as described in E. In D-K, statistics were calculated using the nonparametric Mann–Whitney test. *p < 0.05. n = 5 L4 animals, with 1218 junctions and 1085 tips. n = 5 young-adult animals, with 1610 junctions and 1410 tips. Bars show the mean value and error bars show the standard deviation. (PDF) [file pcbi.1009185.s007.pdf]
