# Supplementary material for: Neuron tracing and quantitative analyses of dendritic architecture reveal symmetrical three-way-junctions and phenotypes of git-1 in C. elegans
Source: PLoS Comput Biol. 2021 Jul 19;17(7):e1009185. doi: 10.1371/journal.pcbi.1009185 (PMC8321406; doi:10.1371/journal.pcbi.1009185)
Supplement: S1 Table — (DOCX) [file pcbi.1009185.s008.docx]

| **Category** | | **Parameter** | **Value** | **Unit** |
| --- | --- | --- | --- | --- |
| Neuron Tracing | Convolution | Rotation range | [-70,70] | ^o^ |
|  |  | Rotation step | 5 | ^o^ |
|  |  | Minimum peak distance | 15 | ^o^ |
|  |  | Minimum peak prominence | 0.4 |  |
|  |  | Forward increment length | 1 | pixels |
|  |  | Normalization minimum peak height | 0.07 |  |
|  |  | Normalization minimum peak distance | 30 | ^o^ |
|  |  | Smoothing parameter | 0.05 |  |
|  | Rectangle size | Rectangle length-width ratio | 2 |  |
|  |  | Rectangle width smoothing parameter | 0.5 |  |
|  |  | Rectangle width sliding window | 6 | steps |
|  |  | Rectangle width scanning resolution | 0.035 | µm |
|  |  | Maximum distance from skeleton segment | 1.5 | µm |
| Preprocessing | CNN | Input_size | 64x64 | pixels |
|  |  | Dataset size (number of input samples) | 15,000 |  |
|  |  | Number of source PVD images | 3 |  |
|  |  | Number of input samples per image | 5000 |  |
|  |  | Training set ratio | 0.8 |  |
|  |  | Solver | Adam |  |
|  |  | Maximum number of epochs | 100 |  |
|  |  | Mini batch Size | 128 |  |
|  |  | Initial learning rate | 0.001 |  |
|  |  | Learning rate drop factor | 0.9 |  |
|  |  | Learning rate drop period | 5 | Epochs |
|  |  | Shuffle | once |  |
|  |  | Filter size | [3,3] |  |
|  |  | Number of convolution layers | 2 |  |
|  |  | Depth | 3 |  |
|  |  | L2 Regularization | 0.0005 |  |
|  | Vertex Convolution | Rectangle length | 1.8 | µm |
|  |  | Circumference scanning resolution | 1 | ^o^ |
|  |  | Minimum peak distance | 20 | ^o^ |
|  |  | Minimum peak width | 5 | ^o^ |
|  |  | Minimum peak prominence | 0.15 |  |
|  |  | Smoothing parameter | 0.99 |  |
| Feature Extraction | Curvature | Smoothing parameter | 0.01 |  |
|  |  |  |  |  |
|  |  |  |  |  |
|  |  |  |  |  |
|  |  |  |  |  |
