# Supplementary material for: Neuron tracing and quantitative analyses of dendritic architecture reveal symmetrical three-way-junctions and phenotypes of git-1 in C. elegans
Source: PLoS Comput Biol. 2021 Jul 19;17(7):e1009185. doi: 10.1371/journal.pcbi.1009185 (PMC8321406; doi:10.1371/journal.pcbi.1009185)
Supplement: Attachment — Submitted filename: Yuval_2020_Response_Reviewers.pdf [file pcbi.1009185.s010.pdf]

Neuron tracing and quantitative analyses of dendritic architecture reveal symmetrical three-way-junctions and phenotypes of *git-1* in *C. elegans*

Detailed response to reviewer comments

Reviewer #1

In this manuscript, Yuval et al. present a novel approach to study the morphology of dendritic spines, and specifically focus on the C. elegans PVD neuron, which has a quite complex structure. The authors combine a CNN with a tracing algorithm to detect PVD dendrites in a manner much superior than other traditional approaches, such as segmentation and skeletonization. The authors show their system performs very well at tracing this complex neuron. In addition, this work presents quantification metrics that describe the spatial arrangement of dendrites with a mathematical basis. Using this tool, the authors characterize in great length the arrangement of PVD in young animals, and then compare these results to mutants of git-1, which is presented as a regulator for dendritic spines. This work is a very methodical, well-thought approach to PVD characterization, which could be used for analysis of many neuron types. It could certainly be used in many different contexts, to study mutant, disease, or aging phenotypes, and thus shed light on the biological mechanisms that control dendritic structure formation. I believe this article topic and quality is suitable for this journal, after the following few items have been addressed.

We thank the reviewer for the positive assessment of our work. In the following, we address the comments and points raised.

1. It is unclear why background subtraction is performed after the active contour model. It would appear that the CNN algorithm already performs this task. Can the authors clarify this point?

We thank the reviewer for this comment. Importantly, the active-contour fitting module of the algorithm operates on the raw, uncorrected microscopy data - not the CNN output. During the fitting stage, rectangular elements are fitted to detect a signal against the (varying) background. Therefore, the local background intensity is subtracted in this process. The output of the CNN stage is used for the tracing stage as guidance cues: we enforce a constraint that requires that pixels of the CNN-derived PVD skeleton are contained within each rectangular element. This procedure is intended to prevent "tracing" of non-PVD elements in the images.

Following this comment, we have now clarified these points in the revised manuscript, see pages 6,7, and pages 1,2 of the SI. We have further revised Figure 1D to clarify the description of the tracing process.

2. Are the scores for each rectangle quantified with the binarized mask? And how is the rectangle height determined? (the authors explain how the width is determined but not the height)

Indeed, the scores for each rectangular size/orientation are calculated from application of the rectangular masks. The length/width ratio of the rectangular elements is taken to be constant, represented by the parameter "Rectangle length-width ratio", listed in Table 1 of the SI. In all

calculations, we have used a length/width ratio of 2. We have now emphasized this point in the main text of the manuscript, at page 7.

3. It appears that manual corrections follow the tracing algorithm, which is then followed by re-training of the CNN. Two points here:

a. How frequent were manual annotations necessary? How many iterations did the system go through?

While some false-positive and false-negative tracings are unavoidable, we believe that manual corrections are actually dispensable. In fact, we have performed the analysis described in the manuscript on uncorrected tracings, and have not found a significant difference in the results (data not shown). However, we did employ manual corrections since our goal in this work is to optimally describe the PVD shape. In order to further address this point and to allow the reader to assess the quality of the tracing process, we now include a panel demonstrating the manual corrections applied, in figure S2B.

b. How would this manually corrected traced PVD help retrain the CNN? I could have misunderstood, but it appears the purpose of the CNN is not binarization but denoising? Clarification in the manuscript text would be very helpful.

We thank the reviewer for this point (also raised by Reviewer #2). We have simplified this algorithm and now assign the CNN the straightforward role of classifying the image pixels as PVD/non-PVD. The output of this classification is subsequently used as guide for the tracing process.

The output of the tracing step adds new information, namely which image pixels are contained

within the rectangular fitted elements. This information allows for an optional re-training of the CNN.

We agree that the "denoising" characterization of the CNN is indeed confusing. In the revised manuscript we have updated the architecture of the CNN and assigned it a purely classifier role. As a result, we recalculated the data for figures 5-6 (we found no essential change in our results). We have addressed this point in pages 5-6, in the revised figures 1D and 2A, and at pages 2-4 of the SI.

4. I suggest modifying the diagram in 3D, if possible, it is a bit difficult to understand what the angle is measuring.

At the suggestion of the reviewer, we have now included an enlarged and expanded blow-up of the angle in figure 3D.

5. For class clustering: how was this done? This is particularly important, since this classification is used when comparing to git-1 mutants. The largest difference is seen in an increase in class 3 and decrease in class 4, but it is unclear how these are found.

We have defined the classes using a straightforward threshold value for the density of elements in the θ-ϕ phase space of the system. The threshold value was simply chosen as the minimal value resulting in four classes. We have now addressed this point on pages 10 and 14 of the manuscript.

6. Related to point 5, from image 4A and C, it is unclear to me how “green dendrites” in C could

all be at phi=0, if some are clearly 180 degrees away from each other. Could the authors clarify this point? In addition, how is it possible that “green” and “blue” dendrites are located at different azimuthal positions, if these are connected and the “blue” dendrites typically do not project radially.

The classification of entire dendritic segments is according to the most abundant class of the rectangular elements that make up the segment (segments defined linear chain of elements between junctions or tips), so that all elements in a segment are assigned the most abundant class. This point is emphasized on page 10,11 of the revised manuscript.

7. I suggest adding background on phenotypes previously observed on git-1 mutants in C. elegans, are there phenotypic differences already known/studied in this organism?

We thank the reviewer for this suggestion. While literature is sparse on behavioral phenotypes of *C. elegans git-1* mutants, in the revised manuscript we have now included our own experimental results on the effects of *git-1* on *C. elegans* speed and crawling gait, as well as its effect on response to harsh touch stimuli.
This new information has been added to the manuscript in pages 15,16,18,19 and in the new figure 7.

8. It would be interesting to add some discussion on how robust the system is to other phenotypes. For instance, old animals exhibit drastic disorganization of PVD. Would this algorithm work as is, or would re-fitting be necessary.

We believe that the system is indeed robust enough to be used on other phenotypes without requiring any modification.

In order to demonstrate this point, we have now included a study of PVD morphology of worms at the L4 development stage. These new analyses, performed without any adjustment to the algorithm, are included in pages 9-10 of the updated SI, and in the new figure S7. In order to analyze drastic PVD phenotypes such as 5-day-old adults or *eff-1* mutants, it will be necessary to train the CNN and do some additional manual adjustments in the process. We expect that our algorithm will manage to analyze strong phenotypes.

Reviewer #2

This paper shows a nice piece of work at the interface between image processing, statistical analysis and biology.

The authors propose an algorithm for detecting dendritic arborization from C elegan images and some statistical tool to analyze its geometrical and topological properties. Some singificatn differences are shwon between wild type and mutant populations. As such, this paper perfectly fits with PLOS Computational Biology aims.

The paper is well written and the work is of quality. However I have a few remarks before this paper can be considered for publications.

We are grateful to the reviewer for these comments and for the positive assessment of our work.

1) The image processing pipeline is not fully clear for me. At first the CNN is proposed to denoise the image. It is then followed by a binarization and by rectangles matching.

- Later on , the CNN is refered to as a classification step : denoising or classification ?

We thank the reviewer for this important correction. Indeed, the CNN role was not sufficiently clear, and its implementation was unnecessarily convoluted. In the revised manuscript, we have updated the architecture of the CNN as a straightforward classifier. As a consequence, we recalculated all analyses presented (this change had no significant effects on the findings).

We have clarified this point in figures 1D and 2A, and on pages 2-4 of the SI.

- The text also refers to active contour. Where exactly are used active contour ? If the image has already been binarized, active contour seems useless.

We refer to the process of rectangular fitting as a specific implementation of the general concept of active contour. Importantly, fitting the rectangular elements is performed on the raw images, not the binarized output of the CNN.

In fact, the representation of the PVD by a set of connected rectangular primitives does not strictly require the CNN. However, this fitting process is very susceptible to error due to image artifacts and to non-PVD features (e.g. gut granules). The classification by CNN is therefore used to complement and constrain the fitting, by requiring that the fitted rectangular elements do not stray from the CNN-derived skeleton representation of the PVD. In this way, we utilize the strength of both CNN and active-contour approaches.

We have now emphasized in the text at pages 6-7 that the rectangular fitting is performed on the raw images, as well as in figure 1D.

- How is made the binarization : thresholding ? (if yes how is estimated the thrershold).

We have defined the morphological classes using a straightforward threshold value for the density of elements in the θ-ϕ phase space of the system. The threshold value was simply chosen as the minimal value resulting in four classes. We have now addressed this point on pages 10 and 14 of the manuscript.

- Figure 1 and the feedback loop are not clear.

We thank the reviewer for this helpful comment. We agree and have now revised figure 1D. We believe the process is now much clearer.

- Contrast is enhanced using photoshop, then there is also a manual correction performed.

Contrast enhancement is usually performed as standard practice in the lab. However, it is not required for our purpose. In the revised manuscript, multiple images of the same worm were still stitched together in Photoshop, but no contrast enhancements were applied. We have updated the manuscript Materials & Methods accordingly. Our experience shows that even manual corrections are drastically less labor intensive than the use of manual tracing or existing tools (order of minutes or less, in contrast to order of hours). Moreover, we believe that manual corrections may not be necessary at all. Indeed, we have performed separate analysis of an uncorrected tracing and found no substantial differences (data not shown). We have included

manual corrections of the automatic process in the manuscript in order to give the best possible characterization of the *C. elegans* PVD morphology.

We have added a new panel to figure S2A to demonstrate the extent of the applied corrections, as well as a detailed comparison with manual/assisted tracing techniques.

- for denoising, simple filters such a median or anisotropic filtering could be also efficient. Did you try before using CNN ?

As the reviewer stated in an earlier comment, the action of the CNN is best described as classification, rather than denoising. CNN output is highly useful in preventing "false-positive" fitting errors and rejecting non-PVD elements such as gut granules.

We have indeed tested off-the shelf and "classical" image processing approaches and have found that such methods are impractical for a high-throughput analysis of PVD microscopy images. We include these results in the SI of the revised manuscript.

In addition, previous attempts to design specialized image processing techniques for PVD analysis (Greenblum et al, Biomed Eng. Online, 2014) have demonstrated that such methods are useful. However, we found that they are not sufficiently robust for large-scale data extraction and analysis.

- How the CNN was trained ? How many samples in the learning set ?

We have now significantly expanded the SI to describe the architecture and training of the CNN (pages 2-4 of the SI). Briefly, we used manually annotated PVD images from three WT animals. From each image 5,000 64x64 pixel image patches were chosen and randomly re-oriented to be used for the CNN training and test. The animals that were used for the CNN training were not included in the WT dataset of animals used for analysis.

- How is evaluated the dentritic tree detection/segmentation ? compared with classical approaches ?

We have verified the segmentation output both by visual inspection, as well as by comparison of multiple metrics (such as total neuronal length and branch distribution) with those determined by manual tracing or off-the-shelf tools. These comparisons are included in the "Manual Validation" section of the revised SI, page 9.

2) why monte carlo simulation are needed to study angle distribution ? Gaussian mixture models seem appropriate.

Importantly, the small-intermediate-large angles of each three-way junction are not independent of each other. Therefore, classical gaussian mixture models may not be applicable to this scenario.

We have emphasized this point in page 7 of the SI.

3) can you elaborate on this assertion "While this variability of the junction angles allows for

intrinsic junction configurations that may deviate from perfect three-way symmetry, these results demonstrate that the orthogonal arrangement of the PVD dendritic arbors (Fig. 1C and 4A) is not a consequence of boundary conditions imposed by PVD junctions, but is rather determined by factors that align the processes themselves"

We agree that the factors that affect the shape of the three-way junctions may be interesting and important, both in the PVD system, and possibly more generally. We specifically intend to address this major question in a separate study, beyond the scope of this present work.
In earlier studies, we have calculated the ground-state configuration of a membrane junction connecting tubular membranal elements with circular cross-sections. Our model has shown that 1) three-way membranal junctions have a lower energy than four- and five-way junctions, and 2) a symmetric planar configuration is the least energy state of three-way junctions. However, we have not yet calculated the effective resistance of a membranal junction to deviations from the ground state. This calculation would enable us to theoretically predict the distribution of junction angles and compare them with the measured variability. Moreover, in these models we have neglected the effects of both intra-junction cytoskeletal elements, as well as the role of membrane proteins that localize to the junctions. Both theoretical and experimental studies are therefore planned in future works.

Reviewer #3

This work gathers the analyses of morphological data of PVD neurons (wild type and GIT-1 mutants) in C. elegans worms. To achieve this goal, the authors use new quantitative techniques

that can potentially be of interest to the worm community. The proposed new imaging processing methods may also facilitate morphological data analysis of PVD neurons in future high-throughput developmental studies and genetic screens. The manuscript has good presentation, however, it suffers from a slight lack of focus. It is hard for the reader to understand if this is a tools and resources paper, or a more biological oriented piece. In order for the manuscript to be better received by the community it could benefit from 1) better curated reference list; 2) new data analysis that highlights the proposed methods large-scale applicability; and/or 3) more detailed description and understanding of the structure-function relationship of the PVD neurons wild type and mutants alike. Please see my concerns in more detail below:

We thank the reviewer for the helpful suggestions, and address the specific points below

Minor concerns:

1) Some of the citations in the introduction concerning the structure-function relationship in dendrites seem to be from a number of tangential studies that were cited just to bulk up the references. I feel that the emphasis should also be put on the vast literature concerning the structure-function relationship in dendrites in nematodes (e.g. C elegans, Drosophila larva). Finally, there are a number of recent studies that focus on quantitative analysis of dendritic structure-function relationship -very relevant to this work- that are not cited in the present manuscript. I believe that the paper will benefit the field more if these changes are accommodated.

We agree. We have now significantly curated and updated the list of references.

Essential/Major concerns:

2) The authors present an interesting image tracing tool and morphological analysis of PVD dendrite structure. While the methods/techniques presented are not entirely novel, they have been optimised for this system which could benefit future studies in the field. If the authors wished for the present manuscript to be a tools and resources paper, rewriting the paper is needed - mostly results and discussion (and Methods in coordination). That is, the paper should focus on the tool and its usage, and not mainly about the subsequent results. The authors need to be clear about what the new image tracing tool brings forth relative to available “off-the-shelf” tools in the field. A title change should be considered in light of these changes. New datasets (e.g., mutants, time-lapse data) should also be analysed to prove the general applicability of the new tracing and analysis methods. These aforementioned datasets could be available to the community already - there is no need to acquire new data. Tutorials on how to use the tool should also be provided, or other technical aspects required for potential users to apply the method on their own data.

We thank the reviewer for these helpful suggestions.

- Manual tracing using "off the shelf" software is possible for tracing of a small data set; However, we find that the time to annotate a single animal required by such methods is drastically longer (over half an hour per animal), making large-scale studies infeasible.

Importantly, the quantitative analysis of the morphology is much simplified since output of our tracing algorithm is an abstracted dataset of elements.

- While we do not wish to lose focus on the biological significance of our findings (see answer to point #3 below), we have now demonstrated the applicability of our fitting algorithm by applying it to an image set corresponding to the PVD of animals at a different developmental stage (L4). Importantly, no re-training or adjustment of the algorithm was necessary. The outputs of this analysis have been added to pages 9-10 the SI of the revised manuscript, and to the new figure S7.
- We have now included a tutorial for the use of the Neuronalyzer tool with the code distribution.

3) Even though the analysis of the morphology of PVD cells is sound, it is not extensive - there is a vast literature of dendrite morphometrics to quantify such tree-like structures that were not used. Moreover, the authors put very little emphasis on interpreting the functional consequences of such results. As an example, only in page 17, lines 18-23, the authors discuss/provide possible functional/behavioural correlates of the found morphological differences in PVD neurons GIT-1 mutants. From a biological point of view, the importance of the study is not clear. Why are three-way-junctions important for the PVD sensory role? Is there any loss-of-function in PVD neurons GIT-1 mutant due to localized reduction in junctions? What mechanistic insights of the PVD system does the reader get after reading the paper? In case the authors wish to present this work as a biological oriented paper, perhaps they should consider expanding on the behavioral phenotypes of GIT-1 and contrast the structure-function relationship of PVD cells wild type vs

GIT-1.

We thank the reviewer for this suggestion. In the revised manuscript, we have now included our results for motility analysis and harsh-touch experiments of *git-1* mutant animals. These findings are presented in the new figure 7, and are presented and discussed on pages 15,16,18,19 of the revised manuscript.

Concluding, essential concerns 2) and 3) need to be addressed. If the authors wish to rewrite the manuscript to focus more on the tools, then essential concern 2) needs to be extensively addressed. If that is not the case, and the authors prefer to provide a deeper analysis of PVD neurons structure-function relationship, then the points raised in essential concern 3) have to be explored. Ideally, both essential concerns would be addressed, if the authors wish to do so.

We have substantially revised the manuscript in order to address both major concerns raised. We believe the updated paper is much improved, and we are grateful to the reviewer for his/her remarks.

Finally, the link to the computer code is not available:

https://github.com/Omer1Yuval1/Neuronalyzer

We apologize for this technical issue; the GitHub access permissions were not set correctly. The source code is now available at the specified link, without the need for per-viewer authorization.
