# Supplementary material for: Neuron tracing and quantitative analyses of dendritic architecture reveal symmetrical three-way-junctions and phenotypes of git-1 in C. elegans
Source: PLoS Comput Biol. 2021 Jul 19;17(7):e1009185. doi: 10.1371/journal.pcbi.1009185 (PMC8321406; doi:10.1371/journal.pcbi.1009185)
Supplement: Attachment — Submitted filename: Yuval_2020_Response_Reviewers.docx [file pcbi.1009185.s011.docx]

**Neuron tracing and quantitative analyses of dendritic architecture reveal symmetrical three-way-junctions and phenotypes of *git-1* in *C. elegans***

**Detailed response to reviewer comments**

**Reviewer #3**

The authors addressed the majority of the points previously raised. The reference list was updated and new datasets were used to highlight the applicability of the proposed methods to other datasets. Additionally, the authors included results for motility analysis and harsh-touch experiments of git-1 mutant animals. This new analysis greatly increased the reader’s understanding of the structure-function relationship of PVD neurons. Overall, the readability of the manuscript improved substantially. However, a more detailed description of the a link between the structural and functional phenotypes is still missing. Please see my concern in more detail below:

Minor concerns:

1. From a biological point of view, a putative mechanism linking PVD git-1 mutants structural and functional phenotypes is still missing. In case the authors wish to do so, they could consider speculate -based on previous literature- how the observed structural changes in the morphology of the PVD in git-1 worms relates to the observed behavioural phenotypes.

We thank the reviewer for the helpful suggestion. We have now expanded the discussion section to include a discussion of the possible link between the structural and functional phenotypes of git-1 mutants.
